# Optimizing Olive (*Olea europaea*) Leaves as a Sustainable Ruminant Feed: Effects of Chemical Treatments on Nutritional Value and Greenhouse Gas Emissions

**DOI:** 10.3390/ani15050705

**Published:** 2025-02-28

**Authors:** Hajer Ammar, Ahmed Eid Kholif, Moyòsore J. Adegbeye, Uchenna Y. Anele, Tarek A. Morsy, Mohamed M. Abdo, Gouda A. Gouda, Hossam H. Azzaz, Soha Ghzayel, Halimeh Zoabi, Bassam Abu Aziz, Secundino López, Mario de Haro-Martí, Mireille Chahine

**Affiliations:** 1 Laboratoire SPADD, High Agriculture School of Mograne, 1121 Mograne, University of Carthage Tunisia, Zaghouan 1121, Tunisia; 2 Department of Animal Sciences, North Carolina Agricultural and Technical State University, Greensboro, NC 27411, USA or ae_kholif@live.com (A.E.K.); uyanele@ncat.edu (U.Y.A.); 3 Dairy Science Department, National Research Centre, 33 Bohouth St. Dokki, Giza 12622, Egypt; tarekalymo@gmail.com (T.A.M.); m.abdo.nrc.eg@gmail.com (M.M.A.); gagouda@gmail.com (G.A.G.); hosam19583@gmail.com (H.H.A.); 4 Research Centre for Animal Husbandry, National Research and Innovation Agency, Cibinong Science Centre, Jl. Raya Jakarta-Bogor, Cibinong, Bogor 16915, Indonesia; alanspeco@gmail.com; 5 High Agriculture School Le Kef 7119, University of Jendouba, Jendouba 8189, Tunisia; mayar20072011@hotmail.com (S.G.); st_halimeh@yahoo.com (H.Z.); bassamabuazez@yahoo.com (B.A.A.); 6 Laboratoire des Substances Naturelles, National Institute of Research and Physico-Chemical Analysis (INRAP), Sidi Thabet, Ariana 2020, Tunisia; 7 Palestine National Agriculture Research Center Biotechnology Department, Higher Agriculture School of Le Kef, University of Jendouba, National Research Center, Beit Qad Agricultural Station, Jenin 206, Palestine; 8 Biotechnology Department, Ministry of Agriculture, National Agriculture Research Center, Jenin 227, Palestine; 9 Epidemiology Department, Ministry of Agriculture, Jenin 227, Palestine; 10 Instituto de Ganadería de Montaña (CSIC-Universidad de León), Finca Marzanas, 24346 Grulleros, Spain; s.lopez@unileon.es; 11 Departamento de Producción Animal, Universidad de León, 24007 León, Spain; 12 Gooding County Extension, University of Idaho, 203 Lucy Lane, Gooding, ID 83330, USA; mdeharo@uidaho.edu; 13 Department of Animal, Veterinary and Food Sciences, University of Idaho, 315 Falls Ave, Twin Falls, ID 83301, USA

**Keywords:** olive leaves, sodium hydroxide, urea, polyethylene glycol, gas production, greenhouse gas

## Abstract

The purpose of this study was to investigate the effects of treating olive (*Olea europaea*) leaves with sodium hydroxide (NaOH) or urea (both at 4% for 40 d) or supplementing with polyethylene glycol (PEG) at 100 mg/g DM on their nutrient composition, gas production, methane (CH_4_) and carbon dioxide (CO_2_) productions, and fermentation profile. The results showed that NaOH-treated leaves had the lowest organic matter and fiber fractions, while urea-treated olive leaves exhibited the highest crude protein content (*p* < 0.001). Asymptotic gas production was significantly higher (*p* = 0.015) in urea-treated olive leaves compared to NaOH-treated leaves. Methane production was highest (*p* < 0.05) in urea-treated leaves and lowest in NaOH-treated leaves. During incubation, urea-treated leaves produced the highest CO_2_ per gram of degradable neutral detergent fiber (*p* = 0.015). Degradability parameters indicated that PEG supplementation led to the highest DM, neutral detergent fiber, and acid detergent fiber degradability (*p* < 0.05) compared to the untreated and NaOH-treated leaves. Urea-treated leaves produced the highest acetic acid (*p* = 0.016) compared to PEG-supplemented and NaOH-treated leaves, showing no significant difference from the control. Butyric acid levels were higher (*p* = 0.011) in NaOH-treated and PEG-supplemented leaves than in the control and urea-treated leaves. Metabolizable energy (ME) was significantly higher (*p* = 0.013) in urea-treated leaves than in untreated and NaOH-treated leaves, with the latter producing the lowest ME. PEG supplementation resulted in significantly higher microbial protein (MCP) production (*p* = 0.023) compared to NaOH-treated and untreated leaves, while the MCP level in the urea-treated group was comparable to all other treatments. In conclusion, PEG supplementation was the most effective treatment for olive leaves’ degradability and MCP, with urea treatment being the second option. From an environmental perspective, NaOH treatment was the most effective for reducing CH_4_ and CO_2_ productions during the fermentation of olive leaves.

## Simple Summary

The rising costs and limited availability of traditional animal feeds have driven the search for alternative options. Olive leaves, an abundant agricultural byproduct, were studied after being treated with sodium hydroxide, urea, or polyethylene glycol to enhance their nutritional value and assess their environmental impact. Sodium hydroxide treatment effectively reduced fiber content and methane emissions, making it a sustainable choice for reducing greenhouse gases. Urea treatment improved protein content, energy availability, and gas production, offering nutritional benefits but with increased methane and carbon dioxide emissions. Polyethylene glycol proved the most effective for breaking down fiber and boosting microbial protein production, optimizing nutrient utilization. These findings demonstrate the potential of treated olive leaves as a sustainable livestock feed that supports agricultural recycling while mitigating environmental concerns.

## 1. Introduction

The sustainability of agricultural practices has become a key focus in modern animal nutrition, particularly in the Mediterranean region. Olive oil production, a cornerstone of Mediterranean agriculture, generates large quantities of byproducts, including olive leaves. These byproducts are rich in nutrients and bioactive compounds, presenting a promising alternative feed source for ruminants [1,2]. In arid and semi-arid regions, ruminant diets have traditionally depended on fibrous crop residues and shrubs, often facing seasonal availability issues and variable nutritional quality [3,4].

Olive leaves are a significant byproduct of the olive supply chain, generated primarily during pruning and harvesting. Pruning, a critical practice for maintaining tree health and optimizing production, occurs mainly in winter but can also take place in summer to manage shoots and suckers [5,6]. This process produces substantial biomass, with leaves accounting for approximately 25% of the dry weight of pruning residues, contributing to an estimated 11.8 million tons of biomass annually in Europe, a significant portion of which is burned, leading to approximately 23% of carbon dioxide (CO_2_) emissions from olive oil production [6]. Additionally, during mechanical harvesting and milling, leaves, twigs, and other debris are removed from the fruit before pressing, representing 4–10% of the total olive weight and nearly 5% of total olive oil byproducts [7]. Given their substantial volume, repurposing olive leaves can mitigate the environmental impact of olive oil production while providing economic value, particularly as a potential feed resource for livestock. Olive leaves stand out for their high fiber content, polyphenols, and other bioactive compounds. However, their direct use in animal diets is limited by anti-nutritional factors, such as tannins and lignin, which hinder digestibility and nutrient absorption [8]. To address these challenges, various processing techniques, including treatment with sodium hydroxide (NaOH) or urea and the addition of polyethylene glycol (PEG), have been studied across various feed plant species [9,10,11,12]. These treatments improve the nutritional value of treated materials, enhance rumen fermentation, and reduce greenhouse gas emissions, making them a more sustainable and practical feed resource.

Sodium hydroxide and urea are widely used to treat fibrous feed ingredients, as they are safe for animal health and can effectively enhance the nutritional value of low-quality feed [11,13]. Sodium hydroxide treatment breaks the ester bonds between lignin and other compounds, thereby opening up polysaccharides such as cellulose and hemicellulose in plant cell walls for digestion by hydrolytic enzymes [11]. On the other hand, urea serves as a non-protein nitrogen source and improves low-quality forage’s nutritive value [14]. Polyethylene glycol is a polyether with a high ability to form stable complexes with tannins, thereby preventing the binding between tannins and proteins. This property makes PEG a common additive to mitigate the adverse effects of condensed tannins in ruminant diets [15,16,17]. Given the potential benefits of these treatments in improving the value of unconventional feed ingredients, it is essential to apply such chemical treatments to maximize their effectiveness. Zoabi et al. [10] treated almond hulls from Tunisia and Palestine with NaOH or urea, or PEG supplementation and showed that urea treatment decreased methane (CH_4_) production and increased fiber degradability and enhanced ruminal fermentation.

A previous study by Aboamer et al. [18] showed that feeding olive trees byproducts treated with 4% urea as a replacement for berseem hay improved nutrients digestibility and improved milk production performance in Barki ewes. Urea provides ruminants with non-protein nitrogen, supporting microbial protein (MCP) synthesis in the rumen and partially replacing other dietary protein sources [19]. Similarly, Molina-Alcaide and Yáñez-Ruiz [20] highlighted the use of NaOH as a tool to improve olive leaves. However, limited studies have investigated the effects of these treatments in both in vivo and in vitro conditions. Al-Masri [21] reported that adding PEG to olive pruning branches increased rumen microbial nitrogen, enhanced fermentation characteristics, and improved the degradability of DM. This effect was attributed to a reduction in the cell wall constituent of olive byproducts and an increase in total extractable condensed tannins with PEG supplementation [10]. Given the advantages of these various treatments, evaluating and comparing their impacts is necessary to identify the most effective approach for optimizing olive leaves in the rumen environment. Furthermore, no study has simultaneously compared the effects of these three compounds (NaOH, urea, and PEG) on olive leaves or other olive byproducts. This research explores the optimization of the leaves as a sustainable ruminant feed, focusing on the effects of chemical treatments on their nutritional value and greenhouse gas emissions. Given the need for alternative, environmentally friendly feed sources, this study investigates the potential of olive leaves, an abundant but underutilized byproduct, to improve livestock nutrition and reduce CH_4_ emissions. By examining various chemical treatments to enhance digestibility and nutrient availability, the study contributes to advancing sustainable livestock feeding practices and provides valuable insights into the dual benefits of improving feed quality while mitigating the environmental impact of ruminant agriculture.

We hypothesized that the treatments would alter the chemical composition of olive leaves by reducing their fiber concentration and increasing their protein content, thereby enhancing their value as a feed resource for ruminants. Therefore, this study aimed to compare the influence of NaOH or urea treatments, or the supplementation with PEG on the nutritional composition, gas production (GP), fermentation parameters, and degradability of olive leaves. It also seeks to evaluate their potential to reduce CH_4_ and CO_2_ productions, contributing to global efforts to mitigate the environmental impact of livestock production. Results of this research will help unlock olive leaves’ nutritional potential, enhance their ruminal degradability, and support sustainability goals in animal agriculture. By addressing the dual challenges of waste management and resource efficiency, this study gives insights into the role of olive leaves in reducing the ecological footprint of livestock production.

## 2. Materials and Methods

### 2.1. Sampling of Olea europaea Leaves

Samples of *Olea europaea* leaves, a byproduct of pruning, were collected in March from various locations in Jenine, Palestine (Latitude: 32°27′33″ N, Longitude: 35°18′03″ and an elevation of 161 m above sea level). Using scissors, branches and twigs from multiple *O. europaea* specimens were harvested. Following the seasonal pruning, fallen branches from 12 trees in a public area of Jenin were gathered. The sub-products from the four nearest trees were combined to form a single sample. In total, three samples of leaves and fine twigs (diameter < 1 mm) were prepared for this study. Fine branches were cut from the fallen material and transported to the laboratory. In the laboratory, leaves and fine twigs with attached leaves were manually separated from the branches, air-dried, and ground at 1 mm sieve. A total of three samples, each consisting of approximately 90% leaves and 10% thin twigs, were collected and used in this study.

### 2.2. Treatments of O. europea Leaves

Air-dried *O. europaea* leaves (1 kg) were treated with urea or NaOH at 4% (DM basis), or supplemented with PEG at 100 mg/g DM. A solution of 4% urea or NaOH (40 g) dissolved in water was sprayed at the rate of 1 L/1 kg olive leaves, ensuring that each kg contained 4% urea or NaOH. Water was added to adjust the moisture content to a level suitable for the ensiling process and anaerobic fermentation. The treated biomass was then kept in airtight anaerobic conditions. The materials in the polyethylene silo bag were manually compressed to remove as much air as possible and rapidly establish semi-anaerobic conditions and then kept for 40 days at room temperature (on average 27 °C). After this storage time, samples were oven-dried at 55 °C for 48 h, ground, and stored in plastic bags for subsequent analysis and in vitro fermentation.

### 2.3. In Vitro Fermentation and Biodegradation

The in vitro fermentation medium was prepared following the method described by Goering and Van Soest [22]. A reducing solution containing sodium sulfide was added (2 mL) to the buffer immediately before adding rumen fluid. Rumen fluid and fermentation medium were mixed at a 1:4 *v*/*v* and flushed with CO_2_ before being dispensed into incubation bottles. Each 250 mL bottle contained 100 mL of incubation medium, prepared by mixing 20 mL of ruminal inoculum with 80 mL of buffer solution.

Ruminal inoculum was collected from the rumen of three male sheep (42 ± 0.6 kg body weight, 25 ± 3 weeks old) at a local slaughterhouse in Cairo, Egypt in a plastic container to preheat it. Before slaughter, the sheep were fed ad libitum a diet containing concentrates, berseem hay, and rice straw at a 500:400:100 ratio on a DM basis, with free access to water. Rumen contents were collected following the standardized procedure for sampling, storage, and use of ruminal contents as recommended by Fortina et al. [23]. At the slaughterhouse, rumen fluid was obtained within 10 min of the animal’s death. Approximately 250 g of rumen contents were manually sampled and squeezed through a colander into a plastic beaker. This process was repeated until a total of 1000 mL of rumen fluid was collected. Rumen fluid from each sheep was collected in an insulated thermos, combined with fluid from the other sheep, and immediately sealed to preserve normal rumen temperature. It was then transported to the laboratory, where the containers were placed in an anaerobic chamber filled with CO_2_. The ruminal fluid was strained through a double-layered cheesecloth to remove large feed particles, while the particulate materials were squeezed to extract microbes attached to feed particles. The initial pH of the inoculum ranged from 6.8 to 6.9. The time from rumen content collection to the start of incubation in the laboratory was approximately 25–30 min in a climate-controlled room (39 °C). Treatments were tested in two separate incubation runs, each with 3 replicates. Additionally, 2 bottles containing inoculum but no feed (blanks) were included in each incubation run to determine baseline fermentation GP.

A sample of 1 g ± 10 mg of olive leaves (untreated or treated with NaOH or urea or untreated olive leaves supplemented with PEG were placed into filter bags (ANKOM F57; Ankom Technology, Macedon, NY, USA) and then transferred to 250 mL ANKOM bottles (Ankom^RF^ Gas Production System). PEG was not applied to the leaves as a treatment but was added to the incubation medium when untreated leaves were used. The bottle was equipped with an automatic wireless in vitro GP module (Ankom Technology, Macedon, NY, USA) fitted with pressure sensors. Prior to incubation, the glass bottles were flushed with CO_2_ to establish anaerobic conditions. Pressure readings were recorded every 10 min for 48 h, and cumulative pressure values were calculated. Gas pressure data were converted into volume (mL) at standard pressure and temperature. Net GP was determined by subtracting gas volume in the blank bottles. At the end of incubation at 48 h, 5 mL gas samples were collected from bottle headspace through the sampling vent and analyzed using a Gas-Pro detector (Gas Analyzer CROWCON Model Tetra3, Abingdon, UK) to measure the concentration of CH_4_ and CO_2_. The production of CH_4_ and CO_2_ per each g of degradable DM (*d*DM), neutral detergent fiber (*d*NDF), and acid detergent fiber (*d*ADF) was calculated.

### 2.4. Sampling and Analysis of Fermentation End-Products

After 48 h of incubation, the fermentation was halted by placing the bottles on ice for 5 min. The pH of the fermentation was immediately measured using a pH meter. The ANKOM F57 filter bags were washed in water and dried in a forced air oven at 55 °C for 48 h and analyzed for DM, neutral detergent fiber (NDF), and acid detergent fiber (ADF) concentrations as previously reported. The degradation rates of DM, NDF, and ADF were calculated by subtracting the weight of the dried residue from the initial weight of the dried substrate. Total gas CH_4_ and CO_2_ production were expressed relative to the degraded DM, NDF, and ADF after 48 h of incubation.

Exactly 5 mL samples of the fermented supernatant fluid from each bottle were collected in glass tubes to determine ammonia-N (NH_3_-N) and total and individual volatile fatty acids (VFA) concentrations. A 3 mL subsample was mixed with 3 mL of 0.2 M hydrochloric acid solution and preserved for NH_3_-N analysis following the method outlined by the AOAC [24]. Additionally, a 0.8 mL aliquot was combined with 0.2 mL of metaphosphoric acid solution (250 g/L) for VFA analysis [25]. Individual VFA, including acetate (C_2_), propionate (C_3_), and butyrate (C_4_), were analyzed using a gas chromatograph (Thermo Fisher Scientific, Inc., TRACE 1300, Rodano, Milan, Italy) equipped with an AS3800 autosampler and a capillary column (HP-FFAP, 19091F-112; 0.320 mm i.d., 0.50 μm film thickness, and 25 m length; J & W Agilent Technologies Inc., Palo Alto, CA, USA). A standard mixture of known concentrations of individual short-chain fatty acids (C_2_, C_3_, and C_4_) (Sigma Chemie GmbH, Steinheim, Germany) was used to calibrate the integrator.

### 2.5. Chemical Analysis

Samples of *O. europaea* leaves (treated or untreated) were analyzed for their chemical composition. Ash content was determined by incinerating the samples in a muffle furnace at 550 °C for 12 h (method ID 942.05), while crude protein (CP) was measured using the Kjeldahl method (method ID 954.01). Ether extract (EE) was quantified using diethyl ether in Soxhlet extractors (method ID 920.39). The concentrations of ash, CP, and EE were measured according to AOAC [24] methods. Neutral detergent fiber content was analyzed with the use of alpha-amylase and sodium sulfite following the procedure described by Van Soest et al. [26]. Acid detergent fiber content was determined according to AOAC [24] (method ID 973.18). Both NDF and ADF were expressed exclusive of residual ash. Acid detergent lignin (ADL) was measured by removing cellulose from ADF through soaking with concentrated H_2_SO_4_, based on ANKOM Technologies’ recommended analytical methods [24]. Non-structural carbohydrates (NSC; OM-CP-EE-NDF) and organic matter (OM; 1000-ash) concentrations were calculated by difference.

### 2.6. Calculations and Statistical Analyses

To estimate GP kinetic parameters, total GP (mL/g DM) data were fitted using the NLIN procedure of SAS (Version 9.4, SAS Inst., Inc., Cary, NC, USA) according to France et al.’s [27] model y = *A* × [1 − e^−c (t−Lag)^], where *y* is the volume of total GP production at time *t* (h), *A* is the asymptotic GP (mL/g DM), *c* is the fractional rate of GP (/h), and *Lag* (h) is the discrete lag time.

The partitioning factor after 48 h of incubation (PF_48_; mg *d*DM: mL gas) was estimated [28]. The volume of gas produced (mL/200 mg DM) after 24 h of incubation (GY_24_) was then calculated as GY_24_ = mL gas per g DM/g *d*MD. The metabolizable energy (ME; MJ/kg DM) was calculated as described by Menke et al. [29] as ME = 2.20 + 0.136 GP + 0.057 CP. Microbial crude protein production was calculated according to the method described by Blümmel et al. [28] using the equation MCP (mg/g DM) = mg *d*MD − (mL gas × 2.2 mg/mL), where 2.2 mg/mL is a stoichiometric factor that expresses the mg of C, H, and O required for the VFA gas associated with production of 1 mL of gas.

Statistical analysis was performed using the GLM procedure in SAS, employing a completely randomized design with the model Y_ij_ = μ + T_i_ + ε_ij_, where Y_ij_ represents the observation, μ is the population mean, T_i_ is the effect of treatment, and ε_ij_ is the residual error. The treatment levels were three for chemical composition (untreated, urea, or NaOH) and four for in vitro rumen fermentation studies (untreated, urea, NaOH, and PEG). Data of each of the two runs of the same sample of the substrate were averaged prior to statistical analysis. Mean values of each individual run (two runs) were used as the experimental unit.

## 3. Results

### 3.1. Nutrient Composition

**Figure 1:**
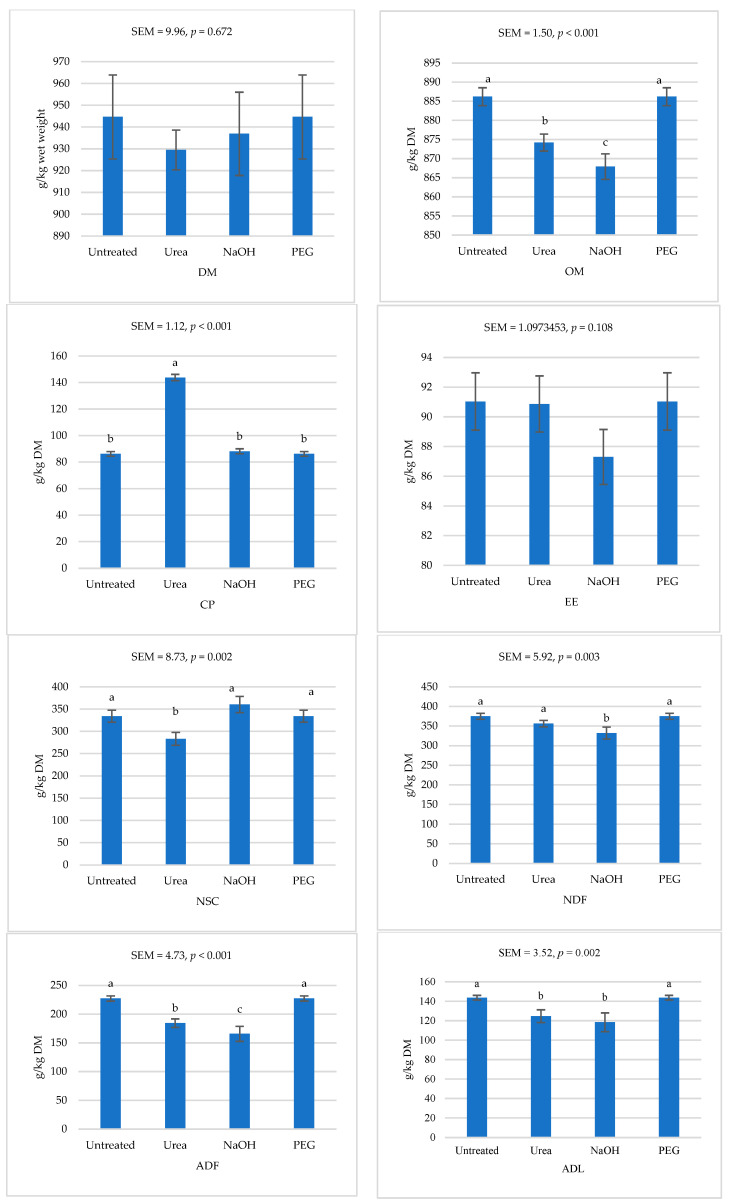
Nutrient concentration of *Olea europaea* (olive) leaves untreated or treated with sodium hydroxide (NaOH), or urea or supplemented with polyethylene glycol (PEG). For each measured nutrient, values in columns with different letters are significantly different (*p* < 0.05). ADF is the acid detergent fiber; ADL is acid detergent lignin; CP is the crude protein; DM is the dry matter; EE is the ether extract; NDF is the neutral detergent fiber; NSC is the nonstructural carbohydrates (OM-CP-EE-NDF); OM is the organic matter (OM; 1000-ash).

Figure 1 illustrates the nutrient concentrations in olive leaves treated with NaOH or urea. Since PEG was only a supplementation rather than a treatment, the PEG-supplemented leaves had similar nutrient concentrations as the untreated leaves. The treatments had no effects on DM and EE concentrations. However, the NaOH-treated leaves, followed by the urea-treated leaves, had significantly lower OM (*p* < 0.001) than the untreated leaves. Olive leaves treated with urea exhibited the highest (*p* < 0.001) CP among the treatments, with no significant differences observed between the other groups. Non-structural carbohydrates were lower in urea-treated olive leaves (*p* = 0.002) than in untreated and NaOH-treated leaves, which showed similar levels. The NDF of NaOH-treated olive leaves was lower (*p* = 0.003) than the untreated leaves. Furthermore, ADF was reduced in NaOH-treated leaves, followed by the urea-treated leaves, compared to the untreated leaves (*p* < 0.001). Acid detergent lignin concentrations were also significantly lower (*p* = 0.002) in both NaOH- and urea-treated leaves than in the untreated leaves.

### 3.2. Gas Production

**Figure 2:**
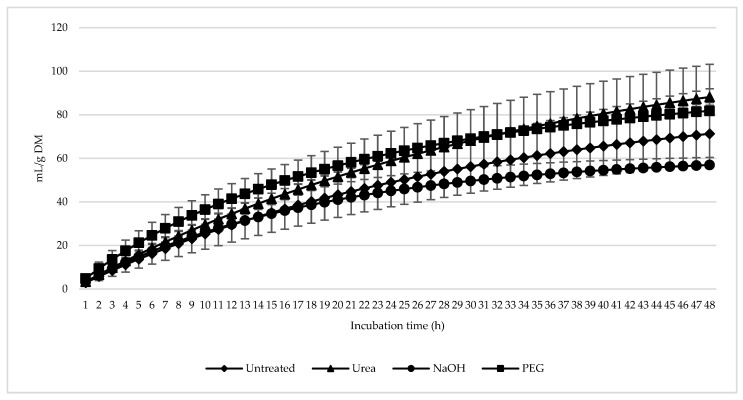
In vitro ruminal total gases production (mL/g incubated DM) of *Olea europaea* (olive) leaves untreated or treated with polyethylene glycol (PEG), sodium hydroxide (NaOH), or urea for 48 h of incubation.

Figure 2 illustrates the trend of the total GP during the 48 h incubation. Table 1 shows the in vitro GP kinetics of olive leaves that were untreated, treated with NaOH or urea or supplemented with PEG. The results indicate that during digestion, the asymptotic GP of urea-treated leaves was significantly higher (*p* = 0.015) compared to NaOH-treated leaves, while no significant differences were observed between the untreated or PEG-supplemented leaves. Moreover, leaves supplemented with PEG or treated with NaOH exhibited higher GP rates (*p* = 0.004) compared to both urea-treated and untreated leaves. The treatments did not affect the lag time or the gas yield at 24 h of incubation.

### 3.3. Methane and Carbon Dioxide Production

Table 2 presents the CH_4_ and CO_2_ production of olive leaves that were untreated, treated with NaOH or urea, or supplemented with PEG. The results show that urea-treated olive leaves produced the highest (*p* < 0.05) CH_4_ across all measures, including mL/g DM, mL/g *d*DM, mL/g *d*NDF, and mL/g *d*ADF during the incubation, while NaOH-treated leaves produced lowest CH_4_. The CH_4_ production per gram of *d*DM, *d*NDF, and *d*ADF was similar among the other treatments. Urea-treated leaves also produced the highest (*p* = 0.015) CO_2_ per gram of *d*ADF compared to NaOH-treated leaves. However, no significant differences (*p* > 0.05) were observed among the treatments in CO_2_ production per g DM, *d*DM, and *d*ADF, nor in the proportion of CO_2_ within the total gas produced.

### 3.4. Fermentation Profile

Table 3 shows the impact of untreated, NaOH-treated or urea-treated, and PEG-supplemented olive leaves on the fermentation profile and digestibility during in vitro digestion. The degradability parameters indicate that PEG supplementation resulted in the highest (*p* = 0.036) *d*DM, *d*NDF (*p* = 0.02) and *d*ADF (*p* = 0.003) compared to untreated and urea or NaOH-treated leaves. Regarding VFA parameters, the treatments did not affect total VFA production, propionic acid level or acetic to propionic acid ratio. Urea-treated leaves produced more acetic acid (*p* = 0.016) than NaOH-treated and PEG-supplemented leaves, with levels similar to the untreated leaves. Butyric acid production was higher (*p* = 0.011) in NaOH-treated and PEG-supplemented leaves compared to the control and urea-treated leaves.

Fermentation parameters revealed that the pH during the incubation was significantly lower (*p* < 0.001) in PEG-supplemented or urea-treated leaves compared to untreated or NaOH-treated leaves. Metabolizable energy was significantly (*p* = 0.013) higher in urea-treated leaves than in untreated or NaOH-treated leaves, with no differences between the PEG-supplemented and untreated leaves. Microbial protein was significantly higher (*p* = 0.023) in PEG-supplemented leaves compared to NaOH-treated and untreated leaves, while MCP produced in the urea-treated group was similar to all other treatments.

## 4. Discussion

### 4.1. Nutritional Characteristics

Crop byproducts and agricultural waste can be effectively used in ruminant diets, as these animals possess a unique ability to digest fibrous crop biomass that monogastric animals struggle to process. Traditionally, ruminants in the Mediterranean region have relied on fodder plants, crop residues, grasses, and pastures as primary forage sources [10]. However, seasonal fluctuations in forage availability make shrubs and tree leaves valuable alternative roughage feeds, particularly in arid regions. These alternatives are particularly beneficial for pastoralists and livestock keepers during drought periods, providing essential crude protein and energy for animal nutrition [30].

One promising fodder plant is *Olea europaea* L. (Olive), a tree widely cultivated in the Mediterranean region. An olive tree can yield approximately 25–30 kg of leaves annually, representing about 5% of its total mass [31]. In the present experiment, since PEG was only a supplementation rather than a treatment, it was added to the untreated leaves just before incubation. Consequently, the PEG-supplemented leaves had a chemical composition similar to that of the untreated leaves. The reduction in OM due to urea and NaOH treatment of olive leaves is attributed to their ability to break down lignocellulosic components, rendering previously bound organic compounds soluble. During alkali treatment, ester and ether bonds are hydrolyzed, breaking down fats and releasing volatile organic compounds [11]. This process converts complex OM into simpler, more digestible forms, enhancing the feed’s nutritional value. NaOH treatment of almond hulls decreased OM, NDF, ADF, and CP concentrations [10].

Crude protein is often deficient in many crop byproducts, and even when present in significant amounts, it is frequently not bioavailable for animal utilization [32,33]. On a DM basis, olive leaves contain 7.0–12.9% CP [34]. In this study, untreated olive leaves contained up to 94.5 g CP/kg DM, which is higher than the values reported by Molina-Alcaide et al. [35] and comparable to those observed by Lee et al. [36]. The increased CP content in urea-treated olive leaves can be attributed to the nitrogen in urea, which serves as a non-protein nitrogen source. This nitrogen can enhance nutritional value directly or indirectly by promoting microbial activity. Urea treatment can improve crude protein content by altering the forage cell wall structure [14].

The treatment effect on NSC was minimal, except in the urea-treated group, where a reduction was observed. This reduction may result from the conversion of urea to ammonia, which reacts with non-structural compounds, reducing their bioavailability. Alternatively, the nitrogen provided by urea may stimulate microbial activity, leading to the consumption of simple sugars and other NSC as an energy source during ensiling or storage, thereby lowering their content.

The observed decreases in NDF, ADF, and ADL in the NaOH-treated group can be attributed to the strong alkali action, which hydrolyzes ester and ether bonds linking lignin to other compounds [37]. This reaction disrupts hydrogen bonds and ester linkages in hemicellulose, breaking it down into soluble sugars and thereby reducing the NDF content of the treated material. Additionally, the crystalline structure of cellulose is partially weakened, resulting in its partial solubilization [11,37]. These findings align with the results of a study by Zoabi et al. [10], which demonstrated that NaOH treatment of almond hulls led to a reduction in NDF and ADF concentrations.

### 4.2. Gas Production

Gas production during anaerobic digestion in vitro serves as evidence of the degradability of soluble substances by ruminal microbes. In this study, the graph of GP showed increased GP for all treatments within 48 h of incubation, reflecting active microbial activity. The higher GP observed in urea-treated olive leaves compared to NaOH-treated leaves underscores urea’s ability to enhance nutrient availability for rumen microbes. Urea is a well-established method for improving the nutritive value of low-quality forage [14,38], as it increases nutrient accessibility and promotes microbial proliferation.

In contrast, the lower GP in NaOH-treated leaves can be attributed to the degradation of simple carbohydrates, fats, and proteins during alkali treatment [10]. The hydrolysis of ester and ether bonds releases volatile organic compounds and soluble phenolics, which are unavailable for microbial fermentation of feeds [39]. Since GP during anaerobic fermentation depends on the availability of degradable carbohydrates [28,40], the reduction in fermentable compounds leads to lower microbial energy production. This effect is further reflected in the reduced ME observed in NaOH-treated olive leaves. Notably, the treatments did not affect lag time and gas yield at 24 h.

### 4.3. Methane and Carbon Dioxide Production

Greenhouse gas emissions from ruminants, particularly CH_4_, are biogenic in origin and an inherent part of their metabolism. As a short-lived climate pollutant, CH_4_ has substantial implications due to its heat-trapping potential and the metabolic cost it imposes on animals. Efforts to mitigate greenhouse gas emissions from livestock are motivated by their impact on climate change, presenting a challenge for animal nutritionists to develop dietary strategies with production efficiency.

In this study, CH_4_ production was highest in urea-treated olive leaves compared to NaOH-treated leaves. Methane production followed a similar trend to the asymptotic GP pattern, as high fermentability releases CO_2_ and H_2_, which serve as precursors for methanogenesis. The reduced CH_4_ output in NaOH-treated leaves likely resulted from the breakdown of organic compounds during treatment, rendering them unavailable for microbial fermentation. These findings are consistent with those of Zoabi et al. [10], Gunaseelan [41], and Liu et al. [42], who also reported reduced CH_4_ emissions from NaOH-treated substrates.

Interestingly, though previous studies have suggested that urea has the potential to reduce CH_4_ production [10], it resulted in the highest CH_4_ in this study. This discrepancy could be attributed to differences in substrate composition. The alkaline nature of NaOH-treated olive leaves may have increased rumen pH compared to urea-treated leaves, inhibiting methanogen activity and thereby further reducing CH_4_ production. Alkali pH can inhibit methanogen activity, since methanogens thrive in a neutral to slightly acidic pH environment. When the pH becomes alkaline, methanogens experience stress, and their activity can be inhibited [43]. This is because high pH can disrupt the cell membrane and enzymatic functions of methanogens, interfering with their ability to reduce carbon dioxide to CH_4_ [43]. While CO_2_ is less potent than CH_4_ or nitrous oxide, it remains a concern due to its extended atmospheric lifespan. As a byproduct of ruminant metabolism, CO_2_ is considered a more favorable alternative to CH_4_ and nitrous oxide.

In this study, CO_2_ production increased with CH_4_ production, with significant differences observed only in CO_2_ (mL/g *d*NDF). This suggests that urea supplementation enhanced fermentation by providing sufficient non-protein nitrogen to rumen microbes, resulting in higher CO_2_ and CH_4_ outputs. Methanogenesis involves the combination of CO_2_ and H_2_, explaining the correlation between GP and CH_4_ production.

Despite the presence of phenolic compounds and condensed tannins in olive leaves [1,36], their inhibitory effect on methanogenesis was minimal, as observed in untreated and PEG-supplemented leaves. Instead, the availability of fermentable substrates and nitrogen played a more critical role in determining GP. The greater availability of substrate and nitrogen, increased GP, along with corresponding increases in CO_2_ and CH_4_ volumes.

### 4.4. Fermentation Profile

Ruminal fermentation enables the host animal to degrade fiber and utilize the end products of microbial fermentation as sources of energy and protein [2]. Modulating this fermentation can improve substrate utilization efficiency, enhancing nutrient conversion to energy and protein for the animal while reducing pollutant emissions [44]. Degradability serves as an indicator of how effectively rumen microbes can break down various components of a substrate.

In our study, PEG-supplemented leaves exhibited the highest *d*DM, while untreated and NaOH-treated leaves showed the lowest. From the proximate analysis, treatments with the highest DM content also had the highest *d*DM, suggesting that the microbial degradation was facilitated by greater nutrient availability. This indicates a potential relationship between substrate composition, availability, and degradability. However, the similar proximate composition of the untreated and PEG-supplemented olive leaves suggests that composition availability alone cannot fully explain degradability. The ability of PEG to bind to tannins and mitigate their inhibitory impact also may have contributed to the increased *d*DM [15,16,17]. The numerical similarity in DM between urea-treated olive leaves and PEG-supplemented leaves, along with comparable *d*DM, indicates that urea improved digestibility by supplying nitrogen to rumen microbes and breaking down complex compounds, thereby making nutrients more accessible.

A similar trend to *d*DM was observed for *d*NDF and *d*ADF. All treatment groups involving treated or supplemented leaves exhibited higher *d*NDF and *d*ADF compared to untreated olive leaves. The common mechanism of the materials used to treat the olive leaves is their ability to break the bonds between compounds [10,11], while PEG binds to condensed tannins, reducing their impact [45]. Additionally, by either breaking down olive leaves’ components or binding specific components, the treatments made cell wall components more accessible for degradation and reduced the activity of phytochemicals such as tannin, condensed tannin, triterpenoids, polyphenols, triterpenes, and other compounds [2,46]. This combined effect of breaking ester bonds within the plant cell wall and reducing phytochemical activity contributed to the increased *d*NDF and *d*ADF in the treatment groups. Furthermore, the similarity in NDF and ADF content between PEG-supplemented leaves and the untreated leaves, alongside the significant differences in their *d*NDF and *d*ADF, highlights PEG’s ability to bind phytochemicals like tannins, reducing their impact and releasing fiber and essential nutrients for degradation. In comparison, the *d*NDF and *d*ADF followed the same patterns of NDF and ADF in the treated leaves. Thus, it can be inferred that PEG treatment reduced phytochemical availability without affecting fiber availability or nutrient content. Conversely, treatments such as urea and NaOH and PEG supplementation reduced both phytochemical activity and fiber content. Supporting this, the similarity observed between *d*NDF and NDF components of treated olive leaves suggests that the degradability of olive leaf components in this study was directly linked to their availability after treatment.

The reduction in acetic acid observed in the urea-treated leaves is linked to the breakdown of complex substances such as cellulose and hemicellulose [10,47]. This breakdown improves nutrient availability and digestibility by enhancing rumen microbial activity, creating a more favorable environment for the specialized microbes. An inverse relationship between acetic acid and butyric acid was observed in this study, with the treatment yielding the highest acetic acid exhibiting the lowest butyric acid levels.

Urea-treated olive leaves had higher acetic acid concentration than NaOH-treated leaves, likely due to the higher GP recorded, indicative of increased microbial activity. Since GP is directly proportional to VFA production [48], the higher GP suggests enhanced fermentation. However, a comparison of acetic acid levels among urea-treated, untreated, NaOH-treated, and PEG-supplemented leaves indicates that GP alone does not fully explain the acetic acid differences. The relationship between acetic acid production and CH_4_ production provides further clarity. During C_2_ formation, one carbon is lost as CO_2_, which can be utilized by methanogens to produce CH_4_. Methanogens in the rumen use H_2_ to reduce CO_2_ and produce CH_4_, maintaining a low partial pressure of H_2_ and driving fermentation toward reduced end products like C_2_ [49]. This interplay between C_2_, CH_4_, and CO_2_ production was evident in both urea-treated and untreated leaves.

The increased butyric acid in the PEG-supplemented and NaOH-treated leaves suggests a shift in fermentation pathways, favoring butyric acid production over acetic acid. This biochemical shift likely contributes to the reduced CH_4_ and CO_2_ production in the PEG-supplemented and NaOH-treated leaves [10]. These findings highlight the potential of PEG and NaOH treatment in altering fermentation dynamics to mitigate greenhouse gas emissions.

Rumen pH serves as an indicator of the alkalinity or acidity of rumen fluid and is influenced by the dietary composition. Previous studies have also shown that dissolved CO_2_ plays a role in determining rumen pH, impacting rumen microbial activity, CH_4_ reduction, VFA proportions, and overall fermentation efficiency [50]. In this study, NaOH-treated and untreated leaves exhibited the highest pH. In contrast, PEG- and urea-treated leaves had lower pH alongside higher GP, indicating that the digestion metabolites may be more acidic than other treatments. This suggests that the metabolites produced during digestion in PEG and urea treatments may be acidic. Notably, the pH of urea-treated and PEG-supplemented leaves is within the optimal range of 6.0–6.8 reported by Kamra [51] and the range of 5.5–7.5 reported for a highly fibrous diet [52].

Metabolizable energy is a key indicator of improved feed fermentation and energy availability, which are critical for meeting the daily energy needs of ruminants [53]. In this study, urea-treated olive leaves had the highest GP and ME, indicating that increased digestibility led to greater animal energy availability. Conversely, the lower GP and ME observed in NaOH-treated leaves suggests that fewer fermentable components were available for microbial digestion. While earlier studies by Khattab et al. [54] and Bachmann et al. [55] reported that urea either did not affect ME or reduced ME, the current findings show that in olive leaves, urea treatment increased ME production. This increase also enhanced MCP, as including urea provided nitrogen in a form readily available to rumen microbes, promoting microbial proliferation and improving GP and degradability [56,57].

The proximate analysis showed urea-treated leaves had higher CP content, contributing to the observed increase in MCP. Although PEG-supplemented leaves had lower CP than urea-treated leaves, they still enhanced MCP. This was likely due to PEG’s ability to bind phytochemicals, allowing rumen microbes better access to nutrients. This effect was evident in the increased *d*DM and *d*ADF observed in PEG-supplemented leaves. Supporting this, Al-Masri [21] reported that PEG addition to olive pruning branches increased rumen microbial nitrogen, fermentation characteristics, and *d*DM by reducing the cell wall constituent and increasing the total extractable condensed tannins. The lower digestibility value observed in untreated and NaOH-treated leaves indicates reduced microbial activity. Furthermore, while high GP is often associated with increased digestibility, it may not always correlate with a beneficial fermentation profile. This is because the gas composition predominantly consists of CH_4_ and CO_2_, which do not directly indicate an optimal fermentation process.

## 5. Conclusions

The study showed that urea-treated and PEG-supplemented olive leaves resulted in higher GP than NaOH-treated leaves, with no significant difference compared to the untreated leaves. Additionally, PEG supplementation resulted in the highest *d*DM, *d*NDF, and *d*ADF. Regarding short-lived climate pollutants, NaOH-treated leaves produced the lowest CH_4_ productions. However, urea-treated leaves had higher acetate and ME, while PEG-supplemented leaves exhibited the highest MCP. Given the combined benefits of reduced CH_4_ and CO_2_ productions, improved degradability, MCP, and C_2_ production, PEG supplementation proved to be the most effective method for processing olive leaves, with urea treatment as a close alternative. This strategy provides a sustainable and efficient way to utilize olive byproducts, particularly in Mediterranean and arid regions. Further in vivo research is recommended to assess the impact of urea-treated and PEG-supplemented olive leaves on animal performance.

## Figures and Tables

**Table 1:** In vitro rumen gas production (GP) kinetics ^1^ of *Olea europaea* (olive) leaves untreated or treated with sodium hydroxide (NaOH), or urea or supplemented with polyethylene glycol (PEG) after 48 h of incubation (*n* = 6 per each mean).

	Treatments ^2^					
	Untreated	NaOH	Urea	PEG	SEM	*p* Value
*A*	93.3 ^b^	61.3 ^c^	121.5 ^a^	95.8 ^b^	13.43	0.015
*c*	0.033 ^c^	0.055 ^b^	0.029 ^c^	0.067 ^a^	0.0025	0.004
Lag	1.68	1.75	1.96	1.78	0.140	0.306
GY_24_	104.2	100.0	116.4	117.3	5.56	0.139

Means in the same row with different superscripts are significantly different (*p* < 0.05). Superscripts were based on the significant *p*-value of the *F*-test for treatment; SEM is the standard error of the mean. ^1^ GP parameters: *A* is the asymptotic GP (mL/g DM), *c* is the GP rate (/h), Lag is the initial delay before GP begins (h), and GY_24_ is the gas yield at 24 h (mL gas/g DMD). ^2^ *Olea europaea* untreated or treated with sodium hydroxide (NaOH treatment), urea (urea treatment), or supplemented with polyethylene glycol (PEG treatment).

**Table 2:** Methane (CH_4_) and carbon dioxide (CO_2_) production ^1^ of *Olea europaea* (olive) leaves untreated or treated with sodium hydroxide (NaOH) or urea, or supplemented with polyethylene glycol (PEG) after 48 h of incubation ^2^ (*n* = 6 per each mean).

	Treatments ^2^					
	Untreated	NaOH	Urea	PEG	SEM	*p* Value
CH_4_ mL/g DM	22.0 ^ab^	13.3 ^b^	25.2 ^a^	21.1 ^ab^	2.81	0.016
CH_4_ mL/g *d*DM	48.8 ^a^	29.5 ^b^	49.8 ^a^	40.0 ^ab^	5.33	0.027
CH_4_ mL/g *d*NDF	53.1 ^a^	28.7 ^b^	55.3 ^a^	43.0 ^ab^	6.58	0.047
CH_4_ mL/g *d*ADF	63.0 ^a^	31.5 ^b^	58.8 ^a^	44.7 ^ab^	7.09	0.029
CH_4_ % of total GP	32.0	23.3	28.7	26.0	2.75	0.222
CO_2_ mL/g DM	46.0	41.9	60.9	58.5	6.39	0.187
CO_2_ mL/g *d*DM	99.3	93.1	120.6	110.2	10.66	0.341
CO_2_ mL/g *d*NDF	108.5 ^ab^	90.7 ^b^	132.0 ^a^	117.9 ^ab^	11.43	0.015
CO_2_ m:/g *d*ADF	129.0	99.4	140.5	123.0	13.25	0.242
CO_2_ % of total GP	65.2	73.6	68.9	71.1	3.01	0.312

Means in the same row with different superscripts are significantly different (*p* < 0.05). Superscripts were based on the significant *p*-value of the *F*-test for treatment; SEM is the standard error of the mean. ^1^ *d*DM is degradable dry matter, *d*NDF is degradable neutral detergent fiber, *d*ADF is degradable acid detergent fiber, GP is gas production. ^2^ *Olea europaea* untreated or treated with sodium hydroxide (NaOH treatment), or urea (urea treatment), or supplemented with polyethylene glycol (PEG treatment).

**Table 3:** In vitro rumen fermentation profile and degradability of *Olea europaea* (olive) leaves untreated or treated with sodium hydroxide (NaOH), urea supplemented with polyethylene glycol (PEG) after 48 h of incubation (*n* = 6 per each mean).

	Treatments ^1^					
	Untreated	NaOH	Urea	PEG	SEM	*p* Value
Degradability ^2^						
*d*DM	465.7 ^b^	450.3 ^b^	505.3 ^ab^	529.0 ^a^	16.6	0.036
*d*NDF	426.7 ^c^	463.0 ^b^	460.7 ^b^	498.0 ^a^	11.4	0.020
*d*ADF	359.7 ^c^	422.3 ^b^	432.3 ^b^	477.7 ^a^	14.3	0.003
VFA ^3^						
Total	24.1	24.0	24.3	24.4	0.10	0.080
C_2_	12.48 ^ab^	10.93 ^b^	12.86 ^a^	10.84 ^b^	0.53	0.016
C_3_	7.92	7.79	7.25	7.97	0.348	0.482
C_4_	3.67 ^b^	5.26 ^a^	4.18 ^b^	5.55 ^a^	0.327	0.011
C_2_/C_3_	1.59	1.40	1.80	1.36	0.141	0. 181
Fermentation ^4^						
pH	6.94 ^a^	6.90 ^a^	6.63 ^b^	6.47 ^c^	0.08	<0.001
ME	4.02 ^bc^	3.93 ^c^	4.61 ^a^	4.39 ^ab^	0.12	0.013
PF_48_	6.64	7.90	5.74	6.76	0.571	0.161
MCP	357.9 ^b^	351.2 ^b^	375.9 ^ab^	392.0 ^a^	7.778	0.023

Means in the same row with different superscripts are significantly different (*p* < 0.05). Superscripts were based on the significant *p*-value of the *F*-test for treatment; SEM is the standard error of the mean. ^1^ *Olea europaea* untreated or treated with sodium hydroxide (NaOH treatment) or urea (urea treatment), or supplemented with polyethylene glycol (PEG treatment). ^2^ *d*DM is dry matter degradability (g/kg incubated), *d*NDF is neutral detergent fiber degradability (g/kg incubated), and *d*ADF is acid detergent fiber degradability (g/kg incubated). ^3^ VFA is volatile fatty acids (mmol/g DM), C_2_ is acetate (mmol/g DM), C_3_ is propionate (mmol/g DM), C_4_ is butyrate (mmol/g DM). ^4^ ME is metabolizable energy (MJ/kg DM), PF_48_ is the partitioning factor at 48 h of incubation (mg *d*DM: mL gas), and MCP is the microbial CP production (mg/g DM).

## Data Availability

The original contributions presented in this study are included in the article, and further inquiries can be directed to the corresponding author.

## Abbreviations

The following abbreviations are used in this manuscript:

*A*	Asymptotic GP
ADF	Acid detergent fiber
*c*	GP rate
C_2_	Acetate
C_3_	Propionate
C_4_	Butyrate
CH_4_	Methane
CO_2_	Carbon dioxide
CP	Crude protein
*d*ADF	Degradable acid detergent fiber
*d*DM	Degradable dry matter
DM	Dry matter
*d*NDF	Degradable neutral detergent fiber
EE	Ether extract
GP	Gas production
GY_24_	Gas yield at 24 h
Lag	Initial delay before GP begins
MCP	Microbial CP production
ME	Metabolizable energy
NaOH	Sodium hydroxide
NDF	Neutral detergent fiber
OM	Organic matter
PEG	Polyethylene glycol
PF_48_	Partitioning factor at 48 h
VFA	Volatile fatty acids
